# Aldosterone Synthase Inhibition in Diabetes and Cardiorenal Metabolic Disease

**DOI:** 10.1111/1753-0407.70253

**Published:** 2026-07-10

**Authors:** Zachary Bloomgarden

**Affiliations:** ^1^ Department of Medicine, Division of Endocrinology, Diabetes and Bone Disease Icahn School of Medicine at Mount Sinai New York New York USA

For clinicians caring for people with diabetes, hypertension, chronic kidney disease (CKD), and heart failure, the renin–angiotensin–aldosterone system (RAAS) should not be considered a single therapeutic axis defined only by angiotensin‐converting enzyme inhibitors (ACEi) and angiotensin receptor blockers (ARBs). The RAAS is a biological network in which angiotensin II, renin, aldosterone synthesis, and mineralocorticoid receptor (MR) activation contribute differently to blood pressure, albuminuria, fibrosis, and cardiovascular‐kidney‐metabolic (CKM) risk. ACE inhibitors and ARBs remain foundational in albuminuric diabetic kidney disease, while beta blockers and direct renin inhibitors illustrate that RAAS‐adjacent pharmacology does not necessarily produce broad cardiorenal‐metabolic benefit. MR antagonists, particularly finerenone, have established aldosterone/MR activation as an outcome‐relevant target in Type 2 diabetes (T2D) with CKD and in heart failure with mildly reduced or preserved ejection fraction. Aldosterone synthase inhibitors, also described as aldosterone‐reducing inhibitors, now add an upstream strategy: reduction of aldosterone production through CYP11B2 inhibition. Recent trials with baxdrostat, lorundrostat, and vicadrostat support clinically meaningful blood pressure or albuminuria effects. The role of aldosterone synthase inhibition should at present be considered complementary to outcome‐proven agents such as ACEi, ARB, beta‐blocker, or MRAs Table 1.

**TABLE 1:** Mechanistic and clinical comparison of RAAS‐related drug classes in diabetes and CKM disease.

Drug class	Principal RAAS effect	Blood pressure reduction	CV outcome	Renal outcome	Diabetes prevention	Anti‐fibrotic	Other effects and cautions
ACEi	ACE inhibition; decreased Ang II generation; increased bradykinin	Moderate. In a 2025 meta‐analysis, standard‐dose monotherapy lowered SBP ~6.8 mmHg from mean baseline SBP 154 mmHg [1]	Outcome‐proven in hypertension, high‐risk vascular disease, post‐MI/HFrEF contexts, and diabetes‐related risk reduction	Basic for diabetic CKD [2, 3]	New‐onset diabetes RR ~0.84 vs. placebo in IPD meta‐analysis [4]	Indirect antifibrotic effect through lower Ang II/AT1 signaling and lower glomerular pressure	Cough, angioedema, hyperkalemia, creatinine rise; avoid ACEi+ARB dual blockade in routine diabetes CKD care
ARBs	AT1 receptor blockade	Moderate. Standard‐dose monotherapy lowered SBP ~8.5 mmHg in the 2025 meta‐analysis [1]	Outcome‐proven in HBP and selected high‐risk CV/renal	Foundational in T2D nephropathy; losartan reduced renal outcomes in RENAAL [5].	Favorable class signal for lower new‐onset diabetes risk; RR ~0.84 vs. placebo [4].	Indirect antifibrotic effects through AT1 blockade.	Better cough/angioedema tolerability than ACEi; hyperkalemia and eGFR monitoring remain necessary.
Direct renin inhibitors	Renin inhibition; decreased Ang I and downstream Ang II generation	Moderate as antihypertensive; limited additive clinical value in high‐risk diabetes when added to ACEi/ARB.	No convincing benefit in high‐risk diabetes or post‐HF [6, 7].	↓Albuminuria; no cardiorenal benefit [6]	No diabetes‐benefit	Theoretical antifibrotic effect not proven	hyperkalemia, hypotension, renal adverse events
Beta blockers	β1 blockade reduces renin release; also sympathetic/cardiac effects independent of RAAS	Moderate. Standard‐dose monotherapy lowered SBP ~8.9 mmHg in the 2025 meta‐analysis [1]	Strong in selected indications: post‐MI, angina, arrhythmia/rate control, and HFrEF depending on agent	No primary diabetic kidney‐protection role comparable to ACEi or ARBs	Less favorable metabolic profile; beta blockers associated with increased new‐onset diabetes risk, RR ~1.48 vs. placebo [4]	Not primarily antifibrotic in diabetes/CKM framing, though selected agents improve remodeling in HFrEF	Bradycardia, fatigue, weight/metabolic effects, masking hypoglycemia; comparator class rather than core RAAS disease‐modifying therapy in DKD
MRAs/aldosterone receptor blockers	MR blockade downstream of aldosterone	Heterogeneous. Spironolactone is strong in resistant hypertension; MRAs averaged ~8.4 mmHg in standard‐dose monotherapy meta‐analysis [1, 8]. Finerenone less BP effects	Finerenone reduced cardiorenal/CV outcomes in T2D CKD and HFmrEF/HFpEF [9, 10, 11, 12]. Steroidal MRAs outcome‐proven in HFrEF	Finerenone reduced CKD progression in T2D CKD; albuminuri reduction class feature [9, 13].	↓new‐onset diabetes FINEARTS‐HF; not yet class‐proven [11].	Strong mechanistic and clinical support; MAGMA linked spironolactone to reduced aortic plaque progression, LV mass, and fibrosis in T2D CKD [14].	Hyperkalemia, eGFR decline, monitoring burden; spironolactone endocrine adverse effects.
Aldosterone synthase inhibitors/ARIs	CYP11B2 inhibition; decreased aldosterone production upstream	Strong emerging signal in uncontrolled/resistant hypertension. Baxdrostat showed placebo‐corrected seated SBP reductions ~8.7–9.8 mmHg; Bax24 showed 24‐h ambulatory SBP reduction ~14.0 mmHg vs. placebo; lorundrostat showed ~7–9 mmHg placebo‐adjusted reductions depending on trial and measurement [15, 16, 17, 18].	Hard CV outcome benefit not yet established.	Albuminuria reduction shown with vicadrostat/BI 690517; CKD outcome role under investigation [15, 19].	Unknown. Metabolic benefit is biologically plausible but unproven.	Plausible through aldosterone reduction; ASI‐specific antifibrotic outcome evidence remains limited.	Hyperkalemia, eGFR dip, hypotension, hyponatremia, adrenal steroid selectivity, and adrenal insufficiency monitoring; may complement proven ACEi/ARB/MRA therapy pending RCTs

## 1 The RAAS in Diabetes and CKM Disease

Diabetes care has shifted from glucose control alone to the prevention of cardiovascular, renal, and metabolic complications as a shared clinical endpoint. The CKM framework captures what clinicians observe daily: Diabetes, obesity, CKD, hypertension, and heart failure do not progress independently, but reinforce one another through hemodynamic stress, sodium retention, inflammation, endothelial dysfunction, and fibrosis [20]. Current diabetes standards continue to place RAAS inhibition at the center of CKD risk management, particularly in patients with hypertension and albuminuria, while adding SGLT2 inhibitors, GLP‐1 receptor agonists, and nonsteroidal MR antagonism to reduce residual risk [2]. The mechanistic question is no longer whether the RAAS matters in diabetes; it is which portion of the RAAS is dominant for a given individual, and how precisely it should be targeted.

This question is of clinical importance in the setting of the movement of aldosterone biology from background physiology to active therapeutic territory. For decades, diabetic kidney disease and hypertension were treated mainly through reduction of angiotensin II signaling. That strategy remains proven. Yet many patients with diabetes and CKD have persistent albuminuria, uncontrolled or resistant hypertension, volume expansion, low‐renin physiology, or heart failure despite ACEi or ARB therapy. In these settings, persistent aldosterone production, aldosterone breakthrough, and MR activation may become clinically important, even when angiotensin blockade is already in place [21, 22].

## 2 Angiotensin II and Aldosterone: Coupled Biology, Separable Targets

Angiotensin II and aldosterone are coupled but have non‐overlapping actions. Angiotensin II, acting mainly through the AT1 receptor, increases vascular tone, promotes efferent arteriolar constriction, supports sodium retention indirectly through aldosterone secretion, and activates inflammatory, oxidative, and profibrotic pathways in the vasculature, myocardium, and kidney [22]. In diabetes, these effects intersect with glomerular hyperfiltration, endothelial dysfunction, adiposity‐related inflammation, sympathetic activation, and vascular stiffness.

The classic action of aldosterone is MR‐mediated sodium reabsorption in the distal nephron, with expansion of intravascular volume and potassium excretion. However, aldosterone‐MR signaling also acts in vascular, cardiac, renal, immune, and adipose tissues, where it is linked to endothelial dysfunction, collagen deposition, macrophage activation, oxidative stress, renal inflammation, podocyte and tubular injury, and myocardial and vascular fibrosis [21]. These mechanisms are particularly relevant to diabetes because CKD, obesity, and insulin resistance often coexist with sodium‐sensitive or low‐renin hypertension. Low‐renin hypertension is common, and renin measurement may increasingly help identify patients whose blood pressure reflects sodium retention or mineralocorticoid biology rather than isolated vasoconstriction [23].

This conceptual approach can be helpful in guiding pharmacology. ACEi reduce angiotensin II generation, though incompletely and with bradykinin effects. ARBs block AT1 receptor signaling. Direct renin inhibitors suppress the top of the cascade. Beta blockers reduce renin release through β1 blockade, while also acting through non‐RAAS cardiovascular mechanisms. MR antagonists block aldosterone receptor signaling downstream. Aldosterone synthase inhibitors, or aldosterone‐reducing inhibitors, reduce aldosterone production upstream by inhibiting CYP11B2.

## 3 Limits of Angiotensin‐Directed Treatment

ACEi and ARBs remain the base layer of RAAS therapy in diabetes because their benefits are supported by kidney and cardiovascular outcome data, not merely blood pressure reduction. Captopril reduced progression in diabetic nephropathy, and losartan reduced renal outcomes in T2D with nephropathy [3, 5]. These and related studies led to current recommendations that ACEi or ARB treatment should be given to people with diabetes and hypertension or albuminuria, with creatinine and potassium monitoring to avoid harm [2, 24].

Studies of direct renin inhibition provide an important caution. Aliskiren lowered blood pressure and could reduce albuminuria, but in high‐risk T2D added to ACEi or ARB therapy it did not improve cardiorenal outcomes and increased adverse events including hyperkalemia, hypotension, and renal complications [6]. ASTRONAUT similarly did not support aliskiren use following hospitalization for heart failure [7]. Recognition that deeper biochemical suppression of the pathway did not improve clinical outcomes, especially in diabetes and CKD where renal hemodynamics, potassium balance, and background therapies strongly condition risk, led to caution in upstream RAAS targeting.

Beta blockers provide a second cautionary comparator. They suppress renin release and lower blood pressure, but their most compelling outcome roles are not in diabetic kidney disease per se, but rather in appropriate settings after myocardial infarction, for angina, rate control, and selected heart failure indications depending on agent and ejection fraction. In uncomplicated hypertension or diabetes‐focused CKM prevention, beta blockers are less attractive as routine first‐line therapy because they lack the albuminuric kidney‐protection evidence of ACEi or ARBs and have less favorable metabolic associations. In an individual‐participant meta‐analysis, ACEi and ARBs were associated with lower new‐onset diabetes risk, whereas beta blockers were associated with higher risk [4].

## 4 MR Antagonism

MR antagonists have supplied the strongest clinical proof that aldosterone/MR activation is more than a blood pressure mechanism. Spironolactone is highly effective in resistant hypertension, consistent with the concept that aldosterone excess or inappropriate sodium retention is a frequent driver of difficult‐to‐control blood pressure [12]. Yet steroidal MRAs are constrained by hyperkalemia, kidney‐function changes, and endocrine adverse effects, especially in diabetes and CKD.

Studies of the nonsteroidal MRA finerenone suggest outcome benefit with MR blockade. In FIDELIO‐DKD and FIGARO‐DKD, finerenone improved kidney or cardiovascular outcomes in T2D with CKD despite leading to only modest blood pressure effects [9, 10]. In FINEARTS‐HF, finerenone reduced the composite of total worsening heart failure events and cardiovascular death in heart failure with mildly reduced or preserved ejection fraction [11]. A prespecified analysis also suggested reduced new‐onset diabetes in participants without diabetes at baseline [12]. These observations suggest that MR activation contributes to cardiorenal‐metabolic disease through inflammatory and fibrotic pathways that are not fully reflected in blood pressure effects. The MAGMA trial extends this concept by linking spironolactone to prevention of aortic plaque progression and reductions in left ventricular mass and fibrosis in T2D with CKD [14].

MRAs therefore define both the promise and the problem for aldosterone‐directed therapy. They demonstrate that the pathway is clinically actionable, but they also show the limitations of downstream receptor blockade. Aldosterone levels can remain high or rise during receptor blockade, MR activation may occur in diverse tissues, and hyperkalemia limits use in precisely the patients most likely to benefit: those with CKD, diabetes, and heart failure.

## 5 Aldosterone Synthase Inhibition Benefits

Aldosterone synthase inhibitors offer a mechanistically different strategy. By inhibiting CYP11B2, they reduce aldosterone synthesis rather than blocking the receptor after aldosterone has been produced. This may be particularly attractive where aldosterone production is inappropriately increased, as in resistant hypertension, low‐renin or volume‐expanded hypertension, primary aldosteronism, and albuminuric CKD. Ongoing studies with this class will show whether aldosterone reduction can provide clinical benefit beyond BP lowering, analogous to the way finerenone has reframed MR blockade.

The blood pressure evidence is now clinically credible. In BaxHTN, baxdrostat added to background therapy reduced seated systolic blood pressure by approximately 9–10 mmHg more than placebo at 12 weeks in uncontrolled or resistant hypertension [16]. In Bax24, baxdrostat reduced 24‐h ambulatory systolic blood pressure by 14.0 mmHg more than placebo in resistant hypertension [25]. Lorundrostat similarly lowered ambulatory systolic blood pressure in ADVANCE‐HTN and automated office systolic blood pressure in the large Launch‐HTN trial, with placebo‐adjusted reductions in the same clinically meaningful range [13, 18]. These effects compare favorably with standard‐dose monotherapy estimates for established antihypertensive classes, although trial populations, baseline blood pressure, background therapy, and measurement method differ [1].

The kidney evidence is earlier but important. Vicadrostat/BI 690517 reduced albuminuria dose‐dependently in CKD on background ACEi or ARB therapy, with similar UACR reductions when added to empagliflozin [19]. Baxdrostat also lowered systolic blood pressure in CKD with uncontrolled hypertension in a population heavily enriched for T2D, though hyperkalemia was frequent [26]. The ongoing EASi‐KIDNEY trial of effects of aldosterone synthase inhibition with vicadrostat beyond blood pressure and albuminuria surrogates toward hard cardiorenal outcomes will lead to greater clarity [15]. Until such outcomes are known, ASIs should be considered promising disease‐mechanism drugs.

Safety will determine clinical positioning. Hyperkalemia, early eGFR decline, hypotension, hyponatremia, adrenal steroid selectivity, and adrenal insufficiency effects will be central to whether ASIs can be layered onto ACEi, ARBs, SGLT2 inhibitors, and MRAs. Baxdrostat and lorundrostat trials have not shown the cortisol‐synthesis effects that limited earlier aldosterone synthase inhibitors, but long‐term adrenal selectivity and real‐world monitoring remain essential [13, 16, 18, 25]. In CKD, the main practical barrier will be hyperkalemia, as it is with MRAs.

## 6 Potential ASI Use Based on Current Evidence

The most likely near‐term use of ASIs is uncontrolled or resistant hypertension, particularly in patients with obesity, CKD, diabetes, or low‐renin physiology. In these patients, ASIs may function as an aldosterone‐production layer added to conventional multidrug treatment. Primary aldosteronism is a specific logical target. The small and open‐label SPARK phase 2a baxdrostat study showed benefit of suppressing aldosterone excess at its source rather than relying only on MR blockade or adrenal surgery [17]. Whether the ASIs will play a role in albuminuric CKD or diabetic kidney disease with residual risk despite standard therapy depends on the outcome of clinical trials.

A major question is whether ASIs will complement or compete with MRAs. MRAs block receptor activation regardless of aldosterone source and have outcome evidence in diabetic CKD and heart failure. ASIs reduce aldosterone production and may be especially useful when excessive aldosterone is demonstrable or suspected. Combination therapy is theoretically potent but may be limited by development of hyperkalemia and renal insufficiency. Appropriate clinical trial evidence will guide their use as additions, based on biomarkers, or as alternatives for patients who cannot tolerate MRAs.

For clinicians in diabetes, practical use should be conservative. ACEi or ARBs remain foundational in albuminuric CKD. SGLT2 inhibitors and GLP‐1 receptor agonists are indispensable CKM therapies even though they are outside classic RAAS pharmacology. Finerenone is the current outcome‐proven aldosterone/MR‐directed therapy in T2D with CKD and now has heart failure evidence in certain settings. ASIs should be watched as a potential next layer for aldosterone‐driven hypertension and residual albuminuric risk. Their future depends on whether aldosterone reduction improves kidney failure, heart failure, cardiovascular death, and tolerability in clinical populations.

## 7 Conclusion

The next phase of RAAS therapeutics in diabetes should not be framed as “more blockade” but as better matching of pathway biology to phenotype. Angiotensin II, renin, aldosterone synthesis, and MR activation each contribute to blood pressure, fibrosis, cardiovascular disease, and kidney disease, but they do so with different therapeutic implications. ACEi and ARBs remain the base layer; beta blockers and direct renin inhibitors remind us that mechanism alone is insufficient; MRAs prove that aldosterone/MR activation is clinically important; and ASIs introduce the possibility of reducing aldosterone at its source. The central test for aldosterone synthase inhibition is whether it can move from compelling mechanism and blood pressure efficacy to durable cardiovascular and renal protection in diabetes and CKM disease.

## Funding

The author has nothing to report.

## Conflicts of Interest

The author declares no conflicts of interest.
